# The emerging role of T cells in depression

**DOI:** 10.3389/fpsyt.2026.1780383

**Published:** 2026-03-23

**Authors:** Huaibing Wang, Hongxia Tao, Minlan Yuan, Wei Zhang

**Affiliations:** Mental Health Center of West China Hospital, Sichuan University, Chengdu, China

**Keywords:** depression, immunotherapy, neuroinflammation, neuroplasticity, T cells

## Abstract

Depression is increasingly recognized as a disorder involving immune brain interactions beyond classical monoaminergic dysfunction. Among immune components, T cells have emerged as key regulators linking peripheral immune dysregulation to central neuroinflammation and impaired neuroplasticity. Accumulating clinical and preclinical evidence indicates that alterations in T cell subsets, including regulatory T cells, Th1 cells, and Th17 cells, contribute to depressive pathophysiology through coordinated effects on blood-brain barrier permeability, glial activation, cytokine signaling, and neurotrophic support. This review synthesizes current evidence on the mechanisms by which T cells migrate into the central nervous system and modulate depressive behaviors. Particular emphasis is placed on the T cell regulation of brain derived neurotrophic factor signaling, and a role for T cell derived extracellular vesicles as modulators of immune neural communication and neuroplasticity. Finally, we discuss the therapeutic implications of targeting T cells in depression, including modulation of T cell subset balance, cytokine-based interventions, microbiota immune regulation, and inhibition of pathogenic T cell trafficking into the brain. Together, these findings position T cells as central orchestrators of immune neural crosstalk and promising targets for mechanism informed immunotherapies in depression.

## 1 Introduction

Depression, a prevalent and debilitating mental disorder, affects an estimated 5.7% of adults globally, corresponding to over 332 million individuals worldwide (1). Beyond its profound impact on emotional well-being, the condition disrupts social functioning, academic performance, and occupational productivity, with suicide accounting for over 700,000 deaths annually and ranking as the third leading cause of mortality among 15–29-year-olds (1). Despite significant advances in psychiatric care, traditional treatments centered on monoamine neurotransmitter balance fail to achieve remission in nearly two-thirds of patients, highlighting an urgent need to unravel the complex pathophysiological mechanisms underlying depression (2).

In recent decades, the neuroinflammation hypothesis has emerged as a pivotal framework for understanding depression, challenging the long-dominant monoamine theory. Chronic stress, infection, and psychological trauma trigger sustained low-grade neuroinflammation, characterized by overactivation of microglia and excessive release of pro-inflammatory cytokines such as interleukin (IL)-1β, IL-6, and tumor necrosis factor-α (TNF-α) (3, 4). These cytokines disrupt neurotransmitter homeostasis, promote neurotoxic metabolite production via the kynurenine pathway, impair hippocampal neurogenesis, and exacerbate hypothalamic-pituitary-adrenal (HPA) axis dysfunction, forming a self-reinforcing cycle that perpetuates depressive symptoms (3). Notably, this inflammatory cascade is tightly regulated by adaptive immunity, with T cells emerging as key mediators linking peripheral immune dysregulation to central nervous system (CNS) pathology (5, 6).

T cells, the core components of cell-mediated immunity, play multifaceted roles in depression through both peripheral and central mechanisms. Clinical studies consistently demonstrate abnormalities in T cell subsets in patients with major depressive disorder (MDD). Approximately 37% of MDD patients show dysregulated T lymphocyte populations, including reduced proportions of CD4+ naïve T cells, elevated levels of Th1 cells, and an imbalanced Th17/regulatory T cell (Treg) ratio (7). Notably, the latter is positively correlated with the severity of depression (8). Beyond peripheral changes, accumulating evidence confirms that T cells can infiltrate the CNS via the choroid plexus, meninges, and blood-brain barrier (BBB), directly modulating neuroinflammation and neural function (9–11). The gut-microbiota-Th17/Treg axis further expands this regulatory network, as gut microbial dysbiosis can alter T cell differentiation and function, indirectly influencing CNS inflammation via the gut-brain axis (12).

Given the growing recognition of T cells as central players in depression pathogenesis, this review aims to systematically synthesize the emerging evidence on the role of T cell subsets in the disease. We first elaborate on the mechanisms by which key subsets (including Tregs (regulatory T cells), Th17, and Th1 cells) regulate neuroinflammation, neuroplasticity in depression. Subsequently, we discuss potential therapeutic strategies targeting T cells, such as modulating T cell differentiation, restoring the Th17/Treg balance, and targeting gut-microbiota-T cell crosstalk. Finally, we address current challenges, including patient heterogeneity, limited clinical translation, and the need for specific biomarkers, and outline future directions for advancing T cell-centered immunotherapies for depression. By integrating these insights, this review provides a comprehensive overview of T cells as promising targets for improving depression treatment outcomes.

## 2 Mechanisms of T-cell migration from periphery to central nervous system

### 2.1 Structural basis of the BBB

The BBB serves as a critical anatomical barrier regulating T-cell entry into the central nervous system (CNS), and its unique structural and functional features determine the specificity of T-cell migration. The BBB is primarily composed of brain microvascular endothelial cells interconnected by continuous and complex tight junctions (TJs), which inhibit paracellular diffusion of solutes and cells (13, 14). Key components of TJs include claudin-5 (responsible for blocking small molecule diffusion), occludin (regulating calcium transport and TJ stability), and junctional adhesion molecules (JAMs, e.g., JAM-A, JAM-B, JAM-C) (15–17). In addition to TJs, adherens junctions (AJs) formed by Vascular endothelial-cadherin (VE-cadherin) are essential for maintaining endothelial cell adhesion and BBB integrity (18). BBB endothelial cells also exhibit low vesicular activity, reducing transcellular diffusion, and express specific transporters and efflux pumps that maintain CNS homeostasis (14). The BBB microenvironment is further regulated by the neurovascular unit (NVU), which includes pericytes embedded in the endothelial basement membrane, astrocyte endfeet forming the glia limitans, and the parenchymal basement membrane. The glia limitans, composed of astrocyte-derived basement membrane and astrocyte endfeet, acts as a secondary barrier preventing uncontrolled immune cell entry into the CNS parenchyma under physiological conditions (19, 20). Recent studies on the NVU and its role in regulating immune cell infiltration into the CNS further support this functional barrier (21, 22).

### 2.2 Multi-step mechanism of T-cell migration across the BBB

**Figure 1:**
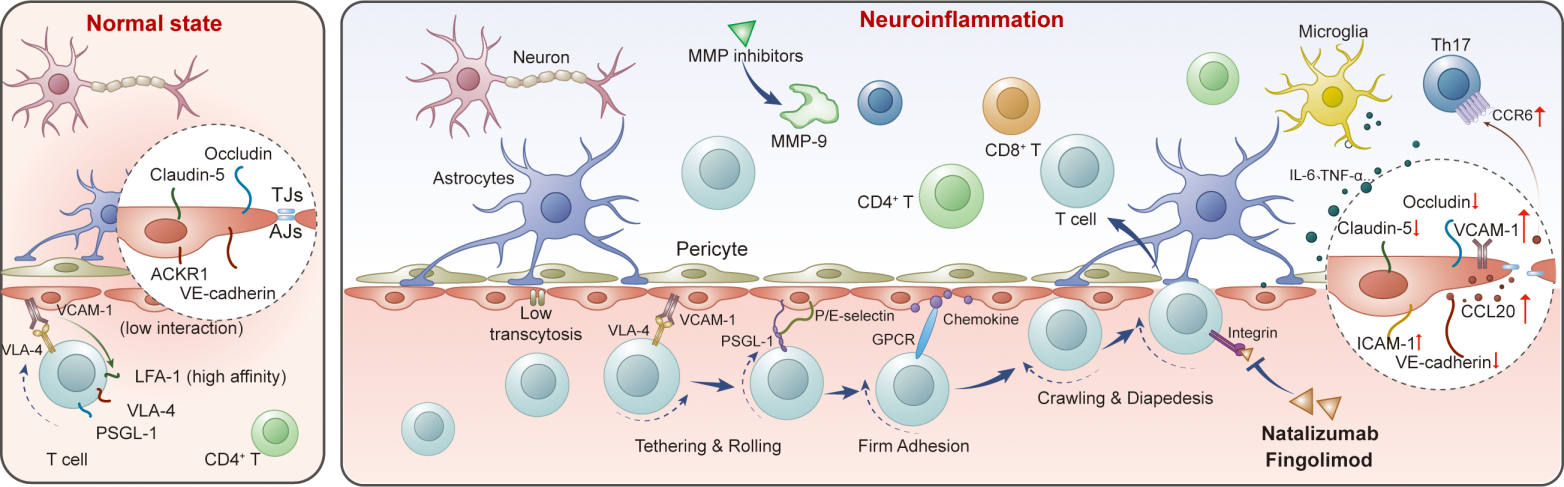
Mechanisms of T-cell migration across the BBB during neuroinflammation. Pro-inflammatory cytokines disrupt tight and adherens junctions, upregulate adhesion molecules (ICAM-1, VCAM-1) and chemokines (e.g., CCL20), and facilitate sequential T-cell tethering, rolling, firm adhesion, crawling, and diapedesis via selectins, integrins, and GPCR signaling. Matrix metalloproteinases (e.g., MMP-9) further enable T-cell penetration across the glia limitans. Distinct T-cell subsets (CD4+, and CD8+) interact with endothelial cells, astrocytes, and microglia to promote neuroinflammation. Therapeutic interventions targeting integrins or MMPs (e.g., natalizumab, fingolimod, MMP inhibitors) can limit T-cell entry into the central nervous system (CNS).

T-cell migration across the BBB follows a highly coordinated multi-step process, which differs significantly between physiological immune surveillance and pathological neuroinflammation (e.g., in depression-associated neuroinflammation) (**Figure 1**) (23, 24). Under steady-state conditions, BBB endothelial cells lack stored P-selectin and constitutively express atypical chemokine receptor 1 (ACKR1), limiting immune cell entry to activated T cells (24, 25). Activated CD4+ T cells are captured via interactions between α4-integrins (e.g., VLA-4, α4β1 integrin) on T cells and vascular cell adhesion molecule 1 (VCAM-1) on BBB endothelial cells, initiating the migration process (26). In the context of neuroinflammation associated with depression, BBB endothelial cells *de novo* express P-selectin, and T cells express P-selectin glycoprotein ligand-1 (PSGL-1). The interaction between PSGL-1 and P-selectin/E-selectin mediates tethering and rolling of activated T cells along the luminal surface of inflamed CNS microvessels, slowing T-cell movement to facilitate subsequent adhesion (27, 28).

After rolling, T cells recognize chemokines displayed on the luminal surface of endothelial cells (or on ACKR1) via G-protein coupled receptors (GPCRs), triggering inside-out activation of integrins (24). This activation enhances the affinity of T-cell integrins for their endothelial ligands:Lymphocyte function-associated antigen 1 (LFA-1) on T cells binds to intercellular adhesion molecule 1 (ICAM-1) on endothelial cells (29, 30).VLA-4 (α4β1 integrin) interacts with VCAM-1 (26, 30). These interactions mediate firm adhesion of T cells to the BBB, a critical step for subsequent transendothelial migration. CD8+ T cells exhibit enhanced dependence on LFA-1 for shear-resistant adhesion and additionally engage endothelial JAM-B, a mechanism not required for CD4+ T-cell adhesion (31–33).

Following firm adhesion, T cells polarize and crawl along the endothelial surface (often against blood flow) to locate permissive diapedesis sites (33, 34). The preferred sites for diapedesis include tricellular junctions and transcellular pathways, with the choice regulated by endothelial ICAM-1 levels and ACKR1 expression. Occurs at bicellular or tricellular junctions, facilitated by interactions between JAMs, PECAM-1, and CD99 (35–37). Low ICAM-1 expression on endothelial cells favors this pathway. Transcellular diapedesis: Involves T-cell penetration through the endothelial cell cytoplasm. High ICAM-1 levels and *de novo* ACKR1 expression during neuroinflammation promote this pathway, reducing T-cell crawling distance and enhancing migration efficiency (25, 34). After traversing the endothelial BBB, T cells enter the perivascular space. To access the CNS parenchyma, they must cross the glia limitans—a process mediated by local pro-inflammatory cytokines (e.g., TNF) that induce expression and activation of matrix metalloproteinases (MMP-2, MMP-9) (38, 39). These MMPs cleave α-dystroglycan (an extracellular matrix receptor on astrocyte endfeet) and modulate chemokine gradients, enabling T-cell penetration into the CNS parenchyma (40).

### 2.3 Regulation of T-cell migration across the BBB in depression

Depression is characterized by low-grade neuroinflammation, which perturbs BBB integrity and modulates T-cell migration-related molecules, creating a microenvironment that facilitates T-cell entry into the CNS (41, 42).

In depressive states, pro-inflammatory cytokines (e.g., IL-6, TNF-α) disrupt BBB tight junctions by downregulating claudin-5, occludin, and VE-cadherin expression (43). This increases BBB permeability and promotes the expression of adhesion molecules (ICAM−1, VCAM−1) and chemokines such as CCL20 on BBB endothelial cells, enhancing immune cell recruitment to the brain (24, 44). The chemokine ligand CCL20 and its receptor CCR6, which are expressed by Th17 cells and endothelial/epithelial cells, constitute a chemotactic axis that facilitates T−cell migration across barrier structures into CNS compartments (44). While this pathway has been well documented in neuroinflammatory conditions like experimental autoimmune encephalomyelitis (EAE), emerging studies in depressive models suggest that Th17 cells and their associated chemokine signaling (involving CCR6 and CCL20, among others) are elevated in inflammatory depression and may contribute to neuroimmune activation and behavioral alterations in depression−like states in rodents and humans (45, 46).ACKR1 mediates chemokine shuttling across the BBB and enhances transcellular T-cell diapedesis during neuroinflammation (25, 47). Genetic variations in ACKR1 may influence T-cell migration efficiency, potentially contributing to individual differences in depression susceptibility (48, 49). α4-integrins (VLA-4) and LFA-1 are critical for T-cell adhesion and migration across the BBB. Immunomodulators such as fingolimod and disease-modifying therapies targeting α4-integrins (e.g., natalizumab) have been shown to inhibit T-cell migration across the BBB, supporting the role of integrins in depression-related neuroinflammation (26, 50). Elevated MMP-9 levels in depression disrupt the BBB and glia limitans, facilitating T-cell entry into the CNS. MMP inhibitors have been proposed as potential adjunctive therapies for depression by reducing neuroinflammation (40, 44).

Different T-cell subsets exhibit distinct migration patterns across the BBB in depression. CD4+ T cells (e.g., Th17, Tregs): Th17 cells rely on CCR6-CCL20 interactions to cross the BBB via the choroid plexus, contributing to neuroinflammation (44). Tregs, while exerting neuroprotective effects, may accumulate in the CNS via α4-integrin-VCAM-1 interactions, and their dysfunction or excessive migration may exacerbate depressive phenotypes (51). CD8+ cytotoxic T cells show enhanced dependence on LFA-1 and JAM-B for BBB crossing (11, 31–33) and, once within the CNS, can directly injure neurons and oligodendrocytes through perforin/granzyme-mediated cytotoxicity, Fas–FasL interactions, and the release of pro-inflammatory cytokines such as IFN-γ and TNF (11). Clinical studies in MDD further demonstrate that altered populations and activation states of cytotoxic T lymphocytes—often together with natural killer cells—are associated with depressive symptom severity, sleep disturbance, and antidepressant response (52, 53), supporting a contribution of CD8+ effector programs to neuroinflammation-related neuronal stress in depression. Their migration is also promoted by neuroinflammation-induced ICAM-1 upregulation (31, 32).

### 2.4 Brain-resident T cells in CNS immune surveillance and depression

In addition to T cells that infiltrate the CNS from the circulation during inflammation, the CNS harbors small but functionally important pools of brain-resident T cells (54–56). These include meningeal and perivascular T cells as well as parenchymal tissue-resident memory T cells that persist long-term within the CNS (54). Under homeostatic conditions, these brain-resident CD4+ and CD8+ T cells continuously survey CNS-derived antigens and maintain local immune surveillance, while generally remaining in a non-pathogenic or regulatory state (54, 57). Through controlled secretion of cytokines such as IL-4, IFN-γ, and IL-17A, brain-resident T cells can influence synaptic remodeling, adult neurogenesis, and cerebrovascular integrity, thereby contributing to the fine-tuning of neuroplasticity and circuit stability (54, 58).

Preclinical studies indicate that chronic stress and systemic inflammation can remodel this brain-resident T cell compartment by altering cell numbers, phenotypes, and cytokine profiles (56, 59, 60). For example, prolonged stress has been associated with an accumulation of brain-resident effector or exhausted T cells, accompanied by enhanced expression of pro-inflammatory cytokines and inhibitory receptors (56, 61). Such changes may lower the threshold for CNS immune activation and promote a shift from physiological immune surveillance toward maladaptive neuroimmune states that impair hippocampal neurogenesis, synaptic connectivity, and stress resilience—core features of depression-related neuroplasticity deficits (62–64). Although direct evidence on brain-resident T cells in patients with MDD is still limited, these findings suggest that local T cell populations in the meninges, perivascular spaces, and parenchyma constitute a critical neuroimmune interface that may participate in the pathogenesis and chronicity of depression and therefore warrant more systematic investigation in future clinical and translational studies.

## 3 Tregs and depression

### 3.1 Inconsistent alterations in T cell proportions/function in depression

Tregs (predominantly CD4+CD25+FOXP3+) exhibit heterogeneous alterations in MDD. Importantly, evidence derives from both clinical studies in humans and preclinical animal models, and these two types of data should be interpreted separately. These inconsistencies can be attributed to various factors, including disease stage, severity, medication status, and comorbidities, which influence Treg dynamics. The complexity of these variables highlights the need for a more refined understanding of Treg involvement in depression.

#### 3.1.1 Disease stage and severity

Treg alterations may differ between acute and chronic depressive states:

Acute depression: In clinical studies involving patients with acute MDD, decreased percentages of circulating CD4+CD25+FOXP3+ Tregs have been reported. Similarly, in acute stress rodent models, transient reductions in splenic or peripheral Tregs have been observed, suggesting that stress-induced immune activation may temporarily suppress regulatory mechanisms (65). This reduction could be linked to an exacerbation of neuroinflammation during the early stages of depression.

Chronic depression: In chronic or recurrent depression, both clinical and preclinical studies report increased Treg frequencies, which may represent a compensatory anti-inflammatory response to prolonged immune activation. In clinical cohorts of patients with recurrent or chronic MDD, some studies also report elevated peripheral Treg counts, possibly reflecting a compensatory anti-inflammatory response (66). The absolute count of CD4+CD25+FOXP3+ Tregs was also significantly higher in the peripheral blood of depressive patients compared to healthy controls, supporting a compensatory increase in Tregs as the body attempts to regulate prolonged inflammation associated with chronic depression (42, 67).

#### 3.1.2 Medication status

The status of medication can profoundly impact Treg alterations in depression:

Antidepressants: Depressive patients receiving antidepressant therapy (e.g., escitalopram, duloxetine, fluoxetine) showed a significant increase in CD4+CD25+ Tregs, suggesting that selective serotonin reuptake inhibitors (SSRIs) and serotonin-norepinephrine reuptake inhibitors (SNRIs) may enhance Treg numbers, potentially mitigating neuroinflammation and improving depressive symptoms (67, 68). Antidepressant treatment likely modulates the immune response by enhancing the regulatory functions of Tregs, reducing inflammation, and improving mental health outcomes.

Unmedicated or severely ill populations: In patients with severe unmedicated depression or post-traumatic stress disorder (PTSD), a relative deficiency in circulating Treg counts has been observed (52, 69, 70). This finding suggests that unmedicated depression or severe PTSD may lead to an impaired immune response, with insufficient Treg function contributing to the chronic inflammatory state that underlies these conditions.

#### 3.1.3 Comorbidities

The presence of comorbid conditions complicates the interpretation of Treg findings:

Older adults: A study involving older adults found that a greater percentage of Tregs correlated with worse physical health status and depressive symptoms, suggesting that Tregs may be involved in a broader context of aging-related inflammation (71). These results suggest that the effects of Tregs may vary based on age and other comorbid factors such as chronic illness.

Sleep disturbance in MDD: Suzuki et al. demonstrated that patients with MDD suffering from sleep disturbance exhibited a significantly increasing trend in CD127+CCR4+ Tregs percentages, implying that disruptions in sleep might exacerbate Treg-mediated immune regulation in depression (53). This highlights the importance of considering the impact of specific comorbidities, such as sleep disorders, on Treg function in depression.

#### 3.1.4 Gut-brain axis and neuroinflammation

An emerging area of research has shown that gut Tregs play a critical role in regulating inflammation and may affect the brain through the gut-brain axis. The downregulation of gut Tregs could lead to an excitatory neuroinflammatory milieu in the brain, contributing to chronic and recurrent stress-induced anxiety and depressive behavior (72). This suggests that dysregulated immune responses in the gut can have profound effects on brain function, further complicating the relationship between Tregs and depression.

#### 3.1.5 Th17/Treg imbalance

**Figure 2:**
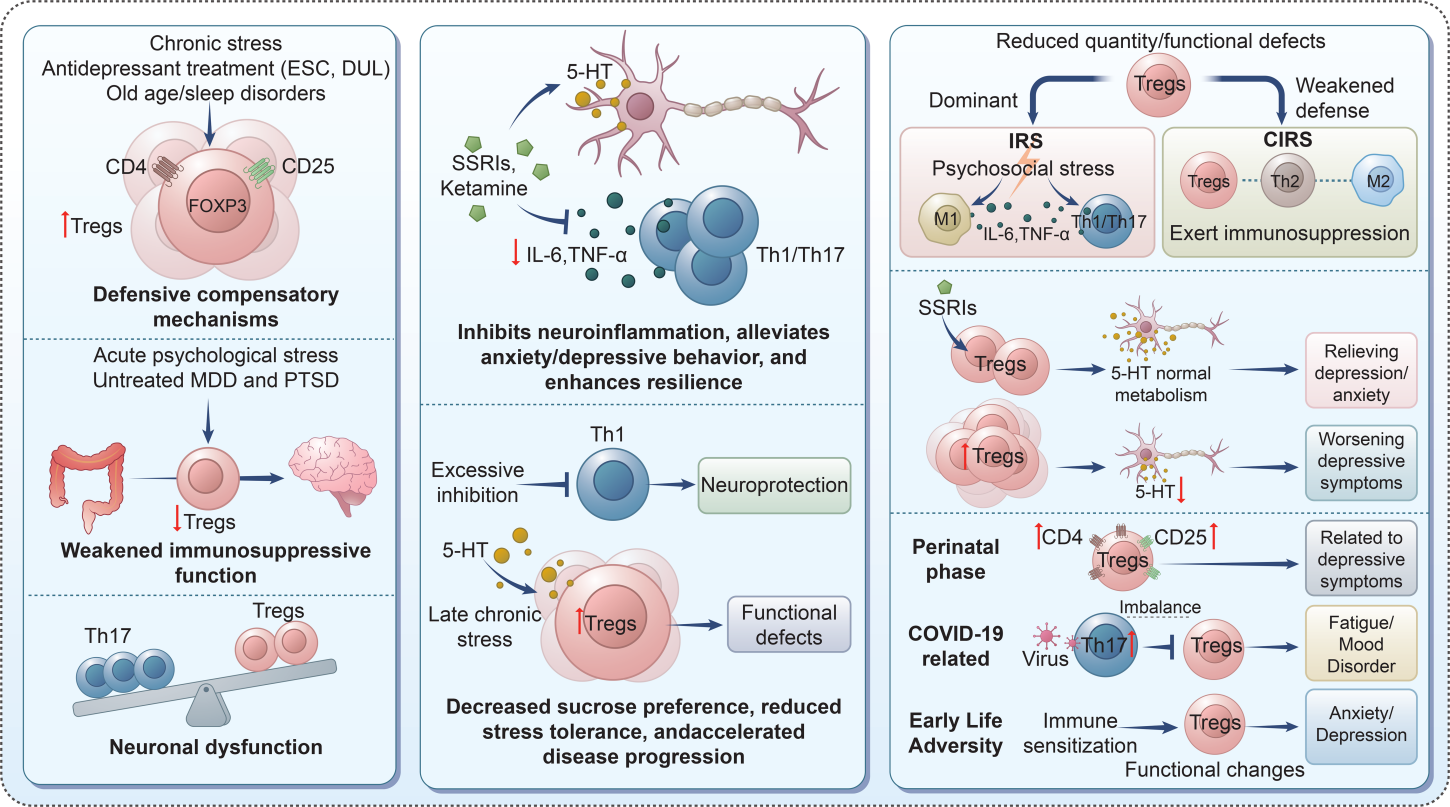
Context-dependent roles of Tregs in stress-related neuroimmune regulation and depression. Chronic stress, antidepressant use, aging, or sleep disorders may induce a compensatory increase in Tregs, whereas acute stress and untreated MDD/PTSD are associated with reduced Treg number or function, leading to Th17 dominance, neuroinflammation, and neuronal dysfunction. Serotonergic signaling and antidepressants (e.g., SSRIs, ketamine) suppress pro-inflammatory cytokines (IL-6, TNF-α) and Th1/Th17 responses, alleviating anxiety- and depressive-like behaviors. However, excessive Treg activation may result in functional defects and impaired stress resilience. Altered Treg balance contributes to immune risk or compensatory immune regulatory states and is implicated in perinatal depression, COVID-19–related mood disorders, and early-life adversity–associated anxiety and depression.

An imbalance in the Th17/Treg ratio is consistently found in depression, with increased Th17 activity contributing to neuroinflammation and neuronal dysfunction. This imbalance plays a crucial role in the pathophysiology of depression, suggesting that Treg dysfunction may drive chronic neuroinflammation and depressive symptoms (**Figure 2**) (73–77).

#### 3.1.6 Treg stability, transcriptional network, and functional plasticity

Beyond quantitative changes in their frequencies, the seemingly contradictory roles of Tregs in depression are likely driven by their stability and functional plasticity in chronically inflamed or stress-related environments. At the molecular level, Treg identity is maintained by a FOXP3-centered transcriptional network rather than FOXP3 alone (78). Stable Tregs are characterized by sustained FOXP3 expression in cooperation with factors such as Helios, Blimp-1 and STAT5 downstream of IL-2 signaling, as well as an epigenetically permissive state at Treg-associated loci (including demethylation of conserved non-coding regions within the FOXP3 locus and accessible chromatin at genes encoding CTLA-4, IL-10, and other suppressive mediators) (78, 79). These features support durable lineage commitment and robust immunoregulatory function.

In contrast, exposure to pro-inflammatory cytokines (e.g., IL-6, IL-1β, IL-23, IL-12, IFN-γ), metabolic stress, or persistent antigenic stimulation can reshape this transcriptional and epigenetic landscape (80–83). Under such conditions, Tregs may up-regulate transcription factors typically associated with effector lineages—such as RORγt in Th17-like Tregs or T-bet in Th1-like Tregs—and progressively lose FOXP3 stability and suppressive capacity. This process generates “ex-Tregs” or functionally unstable Tregs that can produce IL-17A or IFN-γ and fail to control inflammation, despite being phenotypically classified as Tregs in bulk assays (84, 85).

This framework provides a mechanistic explanation for the “dual-edged sword” effect of Tregs observed in depression: in some patients and experimental models, FOXP3-stable, transcriptionally coherent Tregs exert neuroprotective and anti-inflammatory effects, whereas in others, numerically increased but epigenetically destabilized or lineage-skewed Tregs may be ineffective or even contribute to pathogenic immune responses (83, 86, 87). Recent studies on Treg plasticity and FOXP3-network regulation in chronic inflammatory settings support the notion that targeting the mechanisms that stabilize Treg identity—rather than simply increasing Treg numbers—may be critical for harnessing Tregs therapeutically in depression (83, 88).

### 3.2 The relationship between Tregs and depression

Tregs exert anti-inflammatory and immunosuppressive effects, which are considered neuroprotective mechanisms against depressive-like behavior in depression (42, 66). The increased percentages of circulating Tregs may act as a defense mechanism against enhanced inflammatory responses, serving as a pre-indication for chronically stressed individuals (53). The accumulation of Tregs triggered the malfunction of Th1 cells in the brain, leading to cognitive and psychological symptoms, as well as neurodegenerative foci (89). The naturally occurring CD4^+^CD25^+^ Tregs block the endogenous anti-stress neuroprotective effects of T-cells on stress-associated anxiety and depression-like behaviors; a decrease in Tregs improved stress tolerance and mitigated maladaptation to mental stress (90).In the mid-stages of the chronic unpredictable mild stress (CUMS) model, an increase in splenic Tregs accompanied by elevated FOXP3 mRNA expression negatively correlated with sucrose preference (91).Excessive accumulation of Tregs may act as a compensatory factor to counter exaggerated inflammatory responses after prolonged stress, but it can also exert neuropathogenic effects, increasing vulnerability to stressors and contributing to depression progression (89, 90).

Antidepressants can modulate Tregs populations and functions. Mirtazapine, venlafaxine, imipramine, and paroxetine robustly increased the percentages of Tregs in depressive patients (52, 67, 92). SSRIs such as fluoxetine facilitate the expansion of Tregs and their specialized functions, ameliorating neurological dysfunction in postpartum depression (93).Ketamine, a novel antidepressant, rapidly reduces depressive symptoms by influencing the Th17/Tregs balance (94).Acid sphingomyelinase-inhibiting antidepressants (e.g., amitriptyline) induce higher frequencies of Tregs, exerting immunosuppressive effects (95).

### 3.3 The core role of Tregs in depression-related immune homeostasis dysregulation

Depression is accompanied by interconnected inflammatory immune response system (IRS) and counter-inflammatory immune response system (CIRS): IRS is characterized by immune cell activation (M1-like macrophages, Th1, Th17 cells) and pro-inflammatory mediator secretion, while CIRS is dominated by anti-inflammatory properties (Th2 cells, Tregs, M2-like macrophages) (41, 42, 96, 97) Dysregulation of the IRS/CIRS balance due to exogenous psychosocial stress leads to neuroinflammation in depression progression and recovery (42). Weakened or inadequate CIRS defenses (including impaired Treg function) may increase susceptibility to affective disorders (97, 98).

A significant association between increased levels of CD4^+^CD25^+^ Tregs and the severity of depression/anxiety, along with lower concentrations of 5-hydroxytryptamine (5-HT) in depressive patients (70). Huang et al. demonstrated that treatment with Treg promoter IL-2 normalized brain 5-HT metabolism, alleviating depression- and anxiety-like behaviors in the CUMS model (76).

Immune vulnerability is closely associated with depression comorbidity, and Tregs play a critical role by mediating inflammatory responses (99–103).Enhanced CD4^+^CD25^+^ Tregs were found in prenatal and postnatal mothers with depressive symptoms (100).COVID-19 can disrupt the Th17/Treg balance by activating Th17 lineage and repressing Tregs, amplifying inflammation and leading to fatigue, sleep difficulties, and emotional dysregulation (101). Children or adolescents under immune-vulnerable conditions who witnessed adversities may develop internalizing behaviors (anxiety, depression) via immune environment sensitization and altered Treg function (102, 103).

## 4 T helper cells and depression

### 4.1 T helper cell 1/T helper cell 2 imbalance and depression

Despite limited data, increasing evidence suggests that T helper (Th) cells undergo changes in MDD. Some findings include evidence of an increase in Th cells relative to T cytotoxic cells in MDD patients (104, 105) and an increase in the Th1/Th2 cell ratio (106), while antidepressants can reduce this ratio (107).Normally, Th2 is activated by acute mild stress, while Th1 is activated by chronic or recurrent stress (108). In the brains of mice with learned helplessness, the number of Th1 cells increased, although their role in promoting learned helplessness is still unclear (109). However, Th1 cell specific transcription factor Tbet knockout mice did not exhibit depressive like behavior (110), indicating that Th1 cells play a positive role in promoting depressive like behavior.Th1 cells may promote depressive like behavior by activating indoleamine 2,3-dioxygenase (IDO) through the adaptive immune system. In fact, IDO converts tryptophan into canine urea nine, depleting the tryptophan pool required for serotonin synthesis. IDO is activated in depression (111), while antidepressant drugs can reduce Th1 cell levels (112).In addition, predicting severe depression can be predicted by Th1 related genes (113). This indicates that pro-inflammatory Th1 cells promote depression. On the contrary, anxiety like behaviors induced by sepsis related encephalopathy are alleviated by Th2 cells (114). Conversely, TH2 related cytokine IL-4 deficient mice exhibit stronger depression related behaviors (115). IL-4 inhibits depression like behavior induced by IL-1b-mediated astrocyte activation and IL-1b-induced changes in serotonin and norepinephrine levels (116). These pieces of evidence suggest that Th2 cells may have resistance to inflammation and depressive like behavior.

### 4.2 Th 17 cells/Tregs imbalance and depression

Recently, most studies have focused on the newly discovered Th17 cell subset of helper T cells (117). Existing research indicates that Th17 cells are generally increased in patients with depression and have a promoting effect on depressive like behavior. In the blood of patients with severe depression, the level of Th17 cells is significantly elevated (77, 118), and in patients at high risk of suicide, the level of Th17 cells is also significantly elevated (119). Importantly, accumulating evidence suggests that Th17/Treg imbalance in depression may be sex-dependent. Women exhibit a higher lifetime prevalence of depression (approximately 2:1 compared with men) and generally display more robust adaptive immune activation, including enhanced T cell responses and cytokine production (120). Sex hormones, particularly estrogen, modulate CD4+ T cell differentiation in a context-dependent manner and may influence Th17 polarization and Treg stability across the reproductive lifespan (121).

Although Th17 polarization and T cell activation have been reported in several neuropsychiatric conditions, including generalized anxiety disorder (GAD), schizophrenia (SCZ), bipolar disorder (BD), and PTSD, the pattern and magnitude of immune dysregulation differ across diagnoses (122–125). For example, increased Th17-related cytokines and altered CD4+ T cell activation have been observed in subsets of patients with SCZ and BD (126, 127), but these conditions are also characterized by stronger associations with psychosis-related neurodevelopmental and dopaminergic alterations (128, 129). In PTSD, immune dysregulation often reflects stress-induced HPA-axis disruption and glucocorticoid resistance rather than sustained Th17 predominance (130–132). Therefore, while GAD and MDD may share partially overlapping T cell–associated inflammatory mechanisms (133), this does not imply identical pathogenesis. Instead, T cell activation may represent a transdiagnostic vulnerability factor whose clinical manifestation depends on genetic background, developmental timing, hormonal milieu, environmental exposures, and neural circuit susceptibility (134, 135). The emerging transdiagnostic framework of psychiatric disorders suggests that shared immune abnormalities can diverge into distinct clinical phenotypes depending on interacting biological and psychosocial factors (136).

It is worth noting that individuals with Th17 cell related autoimmune diseases have a higher comorbidity rate with major depression (137–139). Notably, Th17-related autoimmune diseases such as systemic lupus erythematosus and multiple sclerosis exhibit a strong female predominance, paralleling the higher lifetime prevalence of depression in women. This epidemiological overlap suggests that sex-specific immune regulation may contribute to disease vulnerability. Females generally exhibit stronger Th17 polarization and more robust adaptive immune activation compared to males (140), which may partly explain the increased susceptibility to inflammation-associated mood disorders in women (121).Consistent with these findings, elevated levels of IL17A and Th17 cell produced inflammatory cytokines were observed in some MDD patients (141–144), but not all (142), as well as in patients with post-traumatic stress disorder (145). In addition, the treatment response of certain antidepressants (combination of bupropion and escitalopram) can be predicted by the level of IL-17A in patients (146). In 40% of psoriasis patients with severe depression, depression related symptoms were alleviated after treatment with anti-IL-17A (147). In psoriasis patients, the use of anti-IL-17ra to block IL-17A receptors and interrupt downstream effectors of IL-17A is associated with an increased risk of mental illness and suicide (148).

Research on rodents has found that IL-17A promotes depressive like behavior in rodents (149), providing evidence for the harmful effects of Th17/IL-17A on depression. Stress can increase IL-17A levels (150–152). Th17 cells increased in the brains of mice with learned helplessness and chronic constraint stress (12, 109, 153).In a mouse model of airway hyperresponsiveness induced by severe allergens, Th2 and Th17 immune responses have been shown to be associated with increased animal fear (154). Stress factors can also significantly affect the expression and functional balance of Th17 cells: when mice are induced to produce stress responses through social failure, the number of Th17 cells in their spleen tissue significantly increases (75); The Th17/Tregs ratio in the liver and ileum tissues of mice under chronic stress showed an increasing trend (155).Th17 cells play a promoting role in the occurrence and development of depressive like behavior, and this conclusion has been fully validated through various experimental methods, including exogenous IL-17A stimulation (149); Using antibodies for IL-17A neutralization intervention (109, 149, 156); Using specific drugs to inhibit Th17 cell function (109); And knocking out Th17 cell specific master transcription factors through genetic engineering methods (109).In mice experiencing cumulative mild prenatal stress, Th17 cells can enhance the activation of microglia in the hippocampus, amygdala, and prefrontal cortex; IL-17 antagonistic therapy can improve depression like and anxiety like behaviors after perinatal stress (156). Moreover, as a key transcription factor, retinoic acid receptor associated orphan receptor gamma T (ROR gamma T) is a necessary condition for Th17 cells to induce depressive like behavior (109, 153).

The possible mechanisms of action of Th17 cells include inducing neuroinflammation and neuronal death by activating microglia and astrocytes [for example, IL-17A can enhance astrocyte mediated glutamate excitotoxicity (157), as well as inhibiting neurogenesis through IL-17A - IL-17A gene knockout mice showed significantly increased neurogenesis levels, which also confirms this mechanism (158).

Th17 cells are present in the lamina propria of the small intestine of healthy mice, which is closely related to the colonization of segmented filamentous bacteria (SFB), which are the key microbiota for inducing Th17 cell differentiation (159). Research has shown that the level of SFB can predict the sensitivity of mice to learned helplessness models through Th17 cell dependence: mice lacking SFB exhibit resistance to learned helplessness (12). Further research has found that in Rag2 ^-^/^-^ mice, SFB specific CD4+ T cells are sufficient to induce depressive like behavior and also promote the aggregation of Th17 cells in the hippocampus (12). The above results suggest that changes in gut microbiota may be an important reason for the enhanced Th17 cell response in depression.

## 5 T cell-mediated regulation of neuroplasticity

### 5.1 T Cell–mediated regulation of BDNF signaling and neuroplasticity

**Figure 3:**
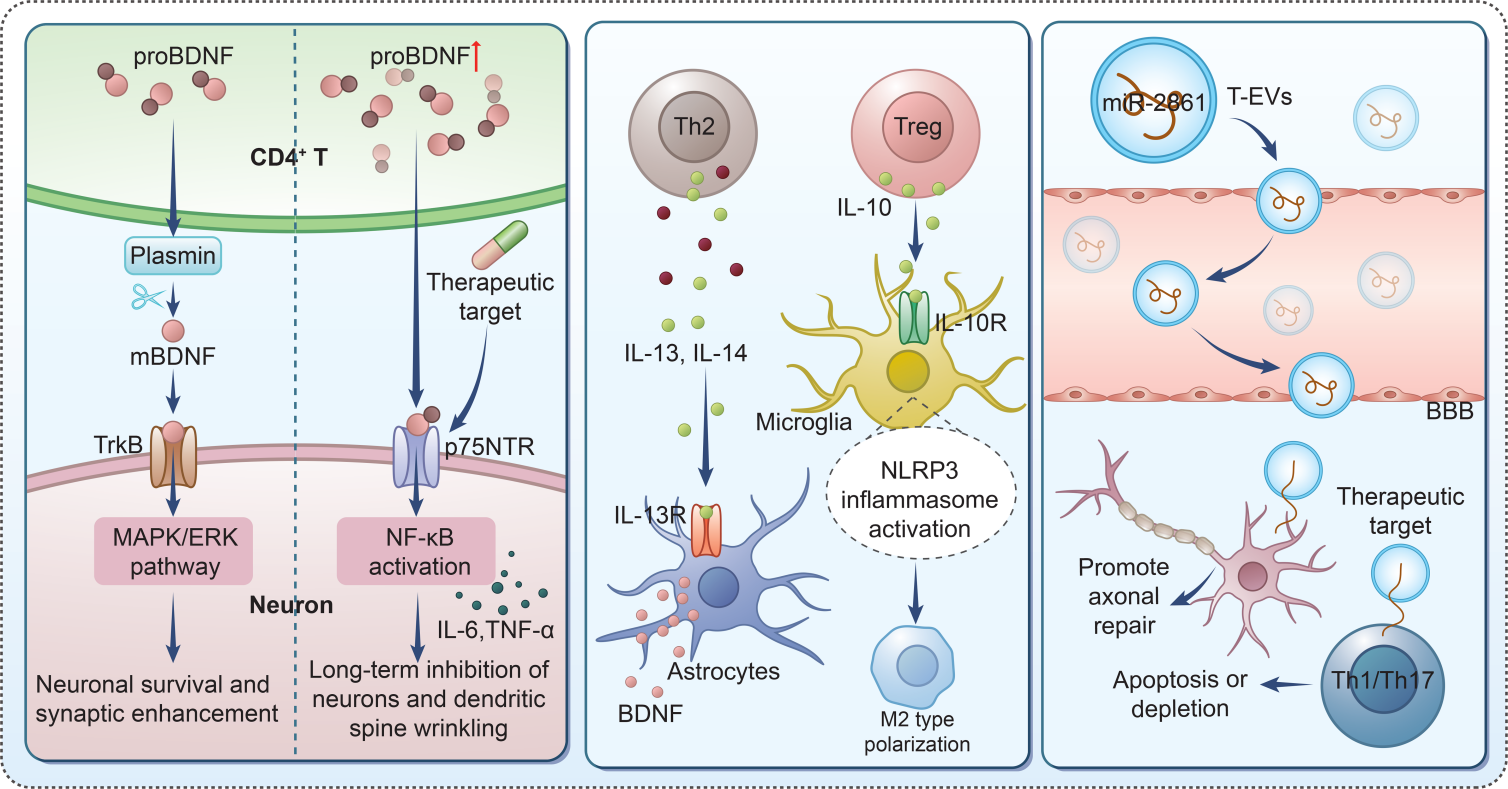
T cell–mediated regulation of neuroplasticity through BDNF signaling and extracellular vesicles.

Neuroplasticity, which includes synaptic remodeling, myelination, and neuronal survival, is a critical target in depression, with brain-derived neurotrophic factor (BDNF) recognized as a key mediator of these processes (160–163). T cells, as central players in the neuroimmune network, regulate neuroplasticity by modulating the balance between brain-derived neurotrophic factor (BDNF) and its precursor proBDNF and influencing downstream signaling pathways (**Figure 3**) (164).

CD4+ and CD8+ T cells directly regulate BDNF and proBDNF expression, impacting neuroplasticity. Clinical studies in MDD show that proBDNF and its receptor p75NTR are highly expressed in peripheral CD4+ and CD8+ T cells, with elevated levels in MDD patients, which normalize after effective therapy (165). Exogenous proBDNF promotes inflammatory cytokine release from peripheral blood mononuclear cells (PBMCs), while blocking the proBDNF/p75NTR pathway inhibits this response (165). Given that proBDNF/p75NTR signaling leads to inflammatory cytokine production in MDD, the dysregulation of T cell-derived proBDNF may impair neuroplasticity by disrupting the inflammatory balance required for synaptic function. In septic mice, proBDNF upregulation in CD4+ T cells suppresses immune activity, reducing CD4+ T cell infiltration into the meninges and downregulating anti-inflammatory cytokines such as IL-4 and IL-13 (166). Since IL-13 stimulates astrocytes to produce BDNF, its reduction may indirectly compromise neuroplasticity. Taken together, these findings indicate that aberrant proBDNF/p75NTR signaling in both CD4+ and CD8+ T cells can couple systemic T-cell dysregulation to impaired BDNF-dependent neuroplasticity in MDD, rather than representing a purely CD4+ T cell–restricted mechanism.

T cells also regulate BDNF expression through interactions with glial cells (astrocytes and microglia), further modulating neuroplasticity. In the CNS, Th2 cells and Tregs collaborate with M2-type microglia to secrete BDNF, promoting neural repair (167). For instance, caloric restriction in EAE mice reduces CD4+ T cell accumulation in the CNS, increases BDNF expression, and enhances myelination, improving neuroplasticity (15). Similarly, human amnion mesenchymal cell (hAMC) treatment reduces CD4+ and CD8+T cell infiltration in the CNS of EAE mice and promotes the production of BDNF in the CNS, thereby alleviating inflammation and promoting myelination (168). In Alzheimer’s disease (AD) models, Aβ-primed astrocytes modulate the cytokine profile of CD4+ T cells, increasing BDNF mRNA levels in CD4+ T cells; this T cell-derived BDNF prevents the reduction of presynaptic protein synaptophysin in neurons through a BDNF-dependent mechanism, protecting synaptic plasticity (169). These findings indicate that T cells can regulate glial cell function and BDNF secretion through cell-cell crosstalk, thereby maintaining the integrity of neuroplasticity.

T cells’ regulation of BDNF-mediated neuroplasticity is closely linked to depression’s pathological mechanisms. Studies on neuropathic pain show that T cell-microglia interactions increase microglial-derived BDNF, which alters neuronal excitability and synaptic transmission, while promoting pro-inflammatory cytokine release from astrocytes (170, 171).

In summary, T cells regulate BDNF-mediated neuroplasticity through multiple mechanisms, including direct modulation of BDNF/proBDNF expression and indirect regulation of BDNF secretion through glial cell interactions. Dysregulation of the T cell-BDNF-neuroplasticity axis may contribute to depression by disrupting synaptic function, myelination, and neuronal survival. Further research into the specific T cell subsets (e.g., Th2, Tregs) and their regulatory effects on BDNF signaling will offer new therapeutic targets for depression, aiming to restore neuroplasticity. Dysregulation of T cell-BDNF pathways, including extracellular vesicle-mediated delivery of regulatory miRNAs, may contribute to impaired neuroplasticity in depression and represents a novel therapeutic target.

### 5.2 T Cells mediate neuroplasticity regulation through exosomes

Exosomes, as key mediators of intercellular communication, are enriched with bioactive molecules such as miRNAs, proteins, and cytokines (172). T cell-derived exosomes (T-EVs) have emerged as critical regulators of neuroplasticity by modulating the neuroimmune microenvironment, balancing immune cell subsets, and delivering functional cargo to target cells (173, 174). Given the close association between neuroimmune dysregulation, impaired neuroplasticity, and depression pathogenesis, T-EVs have become a novel focus for understanding the immune-neural crosstalk in depression.

T-EVs, particularly those derived from Tregs (Treg-EVs), play a pivotal role in maintaining immune homeostasis and promoting neuroplasticity by regulating the balance of pro-inflammatory and anti-inflammatory T cell subsets. *In vitro* studies have shown that exosomes from ex vivo expanded Tregs express Treg-associated markers and suppress pro-inflammatory responses, while intravenous or intranasal administration of these Treg-EVs reduces peripheral and central pro-inflammatory transcripts and expands Treg populations secreting IL-10 and IL-35 (175). This immune modulation creates a tolerogenic microenvironment that supports neuroplasticity, as excessive pro-inflammatory T cell (Th1/Th17) activity is known to impair synaptic function and neural regeneration in depression-related brain regions.

IFNγ-stimulated mesenchymal stem cell-derived exosomes (IFNγ-Exo) further validate the role of T cell subset regulation in neuroplasticity. IFNγ-Exo contains anti-inflammatory RNAs and proteins that inhibit the proliferation of pro-inflammatory Th1 and Th17 cells, reduce the secretion of IL-6, IL-17AF, and IL-22, and induce Treg expansion both *in vitro* and *in vivo (*176). Similarly, PD-L1 and HGF-engineered mesenchymal stem cell-derived exosomes (EXO-PD-L1-HGF) inhibit T cell proliferation, increase the number of CD8^+^CD122^+^IL-10^+^ Tregs, and create an anti-inflammatory microenvironment in ischemic brains, which facilitates neurogenesis and neural repair (177). These findings suggest that T-EVs can indirectly regulate neuroplasticity by skewing T cell subsets toward an anti-inflammatory phenotype, a mechanism potentially disrupted in depression.

Neuroinflammation mediated by activated microglia and astrocytes is a key contributor to impaired neuroplasticity in depression. T-EVs exert anti-inflammatory effects by targeting glial cells and regulating their functional phenotypes. For instance, mesenchymal stem cell-derived exosomes (MSC-EVs) modify the morphology of IFN-γ/TNF-α-stimulated microglia, shifting them from a pro-inflammatory (M1) to an anti-inflammatory (M2) phenotype, which is accompanied by reduced secretion of pro-inflammatory chemokines (e.g., CXCL6, CXCL9) and increased production of anti-inflammatory factors (178). This modulation of microglia function reduces neuroinflammatory damage to neurons and synapses, creating a favorable environment for neuroplasticity.

Astrocytes, as important regulators of synaptic plasticity, also interact with T-EVs to influence neuroplasticity. Astrocyte-derived exosomes can modify the activity of CD4^+^ T cells by regulating the release of IFN-γ, IL-17A, and CCL2 (179), and conversely, T-EVs may modulate astrocyte activation. In Alzheimer’s disease models, exosomes from GABAergic progenitor cells (hiMGEs) inhibit astrocyte activation *in vitro* and *in vivo* by regulating the CD4^+^ Th1 cell-mediated TNF pathway (180), which prevents astrocyte-induced neuroinflammation and preserves synaptic function. For depression, this T-EV-glial cell crosstalk may restore the balance between pro-inflammatory and anti-inflammatory signals in the CNS, thereby reversing neuroplasticity deficits.

Overall, accumulating evidence supports a bidirectional regulatory loop between T cells and glial cells in depression (181). From the T cell side, effector CD4+ and CD8+ T cells secrete cytokines such as IFN-γ, IL-17A, GM-CSF, and TNF, and release extracellular vesicles that polarize microglia toward either neurotoxic or neuroprotective phenotypes and shift astrocytes between pro-inflammatory A1-like and neurotrophic A2-like states (182–184). These glial transitions critically influence synaptic pruning, dendritic remodeling, myelination, and adult neurogenesis, thereby shaping neuroplasticity and stress responsivity (185, 186). From the glial side, activated microglia and astrocytes function as non-classical antigen-presenting cells and as sources of cytokines and chemokines, including IL-1β, IL-6, TNF, IL-33, TGF-β, and CCL2 (187, 188). Through these mediators, glial cells regulate T cell recruitment across CNS barriers, promote the differentiation of Th1/Th17 and cytotoxic programs, or support the expansion and stability of Tregs (188–190). In the context of depression, chronic low-grade activation of this T cell–glia feedback loop is likely to stabilize a maladaptive neuroimmune state characterized by microglial priming, reactive astrocytosis, an imbalance between effector T cells and Tregs, and persistent impairment of neuroplasticity even after the initial stressor has subsided (62, 63, 191, 192).

T-EVs carry a variety of bioactive molecules that directly target neurons and neural progenitor cells to enhance neuroplasticity. Treg-derived exosomes encapsulate miR-2861, which negatively regulates IRAK1 expression to promote the repair of the blood-spinal cord barrier (BSCB) and improve motor function after spinal cord injury (193). The BSCB, similar to the BBB in the brain, maintains CNS homeostasis, and its integrity is critical for synaptic plasticity and neural regeneration—processes often impaired in depression.

Additionally, small extracellular vesicles derived from altered peptide ligand-loaded dendritic cells (A91-Dendritic cell–derived small extracellular vesicles (DsEVs)) induce the activation of Th2 and Tregs, which “home” to lesion areas and increase the release of neurotrophic factors (193). Neurotrophic factors are key drivers of neuroplasticity, promoting axon regrowth, neuron survival, and remyelination.

In depression, the dysregulation of T-EV-mediated neuroplasticity regulation may contribute to disease progression. For example, peripheral T cell reduction and impaired T cell function are observed in neuropsychiatric disorders such as Parkinson’s disease, where neuronal-enriched small extracellular vesicles (NEEVs) suppress T cell activation and differentiation via PD-L1 (193). A similar mechanism may operate in depression, where T-EV dysfunction could lead to unresolved neuroinflammation, reduced Treg activity, and impaired neural repair.

In summary, T cells regulate neuroplasticity through exosomes via multiple interconnected mechanisms: balancing T cell subsets to create a tolerogenic microenvironment, modulating glial cell function to reduce neuroinflammation, and delivering bioactive molecules to promote neural repair and synaptic function. The dysregulation of this T-EV–neuroplasticity axis may be a key component of depression pathogenesis. From a translational perspective, these properties also position T cell–derived exosomes as attractive candidates for minimally invasive biomarkers (for example, circulating EV signatures reflecting neuroimmune activation and treatment response) and as nanoscale drug-delivery vehicles capable of crossing biological barriers such as the BBB. However, current evidence in depression exosome-based diagnostics or therapeutics specifically targeting MDD have not yet been validated in large clinical cohorts or interventional trials. Thus, while T-EVs provide a conceptually promising platform for disease stratification and targeted drug delivery, robust clinical data are still lacking, and further research into the specific cargo of T-EVs in depression, their targeting mechanisms, and their safety and efficacy in humans will be essential for advancing exosome-based therapies to restore neuroplasticity in depression.

## 6 T-cell targeted therapy in depression

### 6.1 Therapeutic potential of Tregs in depression

Tregs have emerged as promising targets for depression treatment due to their critical role in modulating neuroinflammation and immune homeostasis. Several therapeutic strategies targeting Tregs have shown preclinical feasibility and potential for clinical translation. However, the risk of autoimmune reactions and infections with prolonged immunosuppression should be considered when evaluating these therapies.

Adoptive Treg Transfer: Transplantation of ex vivo expanded or engineered Tregs has demonstrated neuroprotective effects in neuroinflammatory disease models, which are highly relevant to depression pathophysiology. For example, adoptive transfer of activated Tregs ameliorated neurotoxic microglial activation in a mouse model of Parkinson’s disease and reduced needle-trauma-induced death of midbrain dopamine neurons, which are also involved in the pathogenesis of MDD (194, 195). However, the risks of long-term immunosuppression, such as increased susceptibility to infections and autoimmune conditions, remain a concern.

CD28 Superagonists, IL-2/IL-2 Antibody Complexes, and IL-33: These strategies have been shown to expand brain-resident Tregs and enhance their immunosuppressive functions, controlling neuroinflammation in CNS disease models. While preclinical studies have demonstrated potential benefits, long-term use of immune-modulating therapies may carry risks, including the possibility of immune system dysregulation and potential autoimmune flare-ups in susceptible individuals (196–198).

### 6.2 Therapeutic potential of Th17 cells in depression

Targeting Th17 cells offers considerable therapeutic potential for depression, with multiple actionable pathways that avoid direct IL-17A neutralization. However, risk assessment in Th17-targeted therapies is essential, particularly concerning immune suppression and the long-term safety of modulating these pathways.

Th17 Cell Modulation: A primary strategy focuses on inhibiting Th17 cell differentiation by targeting upstream regulatory molecules. For example, pharmacological blockade of RORγT—the master transcription factor driving Th17 lineage commitment—has been shown to block Th17-mediated depressive-like behaviors in preclinical models. Similarly, anti-IL-12/IL-23 antibodies (e.g., ustekinumab) improve depressive symptoms in patients with inflammatory disorders by inhibiting IL-23, a cytokine critical for Th17 cell survival (109, 199, 200). Despite these promising results, glucocorticoids, which are often used for their anti-inflammatory properties, have limited utility in MDD due to their inability to suppress Th17 cells effectively. In fact, glucocorticoids can enhance Th17 cell survival, making them resistant to glucocorticoid-induced apoptosis. This resistance underscores the need for alternative approaches targeting Th17 cells without relying on glucocorticoids (201, 202).

Indirect Modulation via Gut-Microbiota-Th17 Axis: Another promising approach involves modulating the gut microbiome to influence Th17 differentiation. Depleting specific gut microbes, such as SFB, which induces Th17 differentiation, confers resistance to learned helplessness in stressed mice (12). Conversely, administering probiotics that promote anti-inflammatory Tregs (e.g., Clostridium clusters IV/XIVa, Lactobacillus, Bacteroides) restores the disrupted Th17/Treg balance and ameliorates depressive-like behaviors (157, 203). Although this approach is non-pharmacological, the long-term effects of manipulating the gut microbiome require careful monitoring, as alterations to the microbiome can have systemic impacts.

Blocking Th17 Cell Trafficking: Th17 cell infiltration into the CNS is a key step in their pathogenicity in depression. Inhibiting CCR6, a chemokine receptor necessary for Th17 cell migration, offers a precision-focused strategy. Preclinical studies have shown that CCR6 deficiency prevents Th17 cell infiltration into the CNS, reducing depressive-like behaviors. However, the risk of disrupting protective Th17 responses in other tissues, such as the gut, must be considered before clinical application (153, 204).

### 6.3 Cytokine-targeted therapies: risks and ethical considerations

Cytokine-targeted therapies have shown promising results in modulating immune responses and potentially alleviating depression symptoms. However, these therapies come with risks, including infections, autoimmune reactions, and long-term safety concerns.

IL-2 and IL-33 Modulation: IL-2 is critical for Treg lineage stability and proliferation, and low-dose IL-2 has been shown to attenuate depression-like behaviors in a chronic stress mouse model by restoring the Th17/Treg balance. Similarly, IL-33 promotes Treg accumulation and function, enhancing neuroprotective effects. While these strategies show potential, they require careful consideration of the risks associated with prolonged immunomodulation, including the potential for immune suppression and the risk of triggering autoimmune conditions (51, 205, 206).

Anti-IL-17A Therapy: Anti-IL-17A monoclonal antibodies have shown efficacy in reducing depressive symptoms in patients with comorbid autoimmune diseases, such as psoriasis. An integrated analysis of three phase 3 clinical studies demonstrated that ixekizumab reduced depressive symptoms by 40% in patients with moderate-to-severe psoriasis and comorbid MDD, associated with decreased systemic inflammation. However, the use of brodalumab (anti-IL-17RA) has raised concerns due to its association with increased suicide risk and psychiatric adverse events, as highlighted by the FDA warning (147, 199). This underscores the importance of carefully assessing the psychiatric risks associated with prolonged immunomodulation, particularly in vulnerable populations.

Ethical Considerations: Long-term immunotherapy for mental illnesses, including depression, raises ethical questions regarding the potential risks and benefits. While these therapies may offer significant benefits in modulating immune responses and alleviating depressive symptoms, they also carry the risk of immune suppression, increased susceptibility to infections, and the possibility of triggering autoimmune reactions (8). The ethical implications of using such therapies in long-term depression treatment must be carefully weighed, considering the potential for side effects and the impact on patients’ overall health and quality of life. It is essential to ensure that these therapies are applied in a controlled manner, with ongoing monitoring for adverse effects.

In summary, the therapeutic potential of Th17 cell modulation and cytokine-targeted therapies in depression holds promise but must be accompanied by careful risk assessments, particularly concerning immune suppression and long-term safety. The failure of some therapies, such as glucocorticoids in Th17-related depression, highlights the need for alternative approaches. Additionally, the ethical implications of long-term immunotherapy, particularly in vulnerable populations, must be considered in the development of these treatments. Further research into the mechanisms of Th17 cells in depression, their resistance to glucocorticoids, and the long-term effects of immunomodulation will be crucial in optimizing these therapies for clinical use.

### 6.4 Therapeutic challenges and limitations

Despite the promise of T cell-targeted strategies in depression, several challenges hinder their clinical translation. First, most evidence derives from preclinical models or autoimmune cohorts, with limited validation in well-phenotyped MDD patients. Second, long-term immunomodulation carries inherent risks, including increased susceptibility to infections, autoimmune reactions, and potential psychiatric adverse events, as seen with agents like brodalumab. Third, individual differences—such as genetic background, disease stage, sex, and microbiome composition—may significantly influence treatment response and tolerability. These factors underscore the need for precision immunophenotyping and cautious risk–benefit assessment in future trial designs.

## 7 Conclusion

Accumulating evidence indicates that depression involves dysregulated immune neural interactions in which T cells play an active regulatory role. Rather than serving as peripheral markers of inflammation, T cells directly influence neuroinflammatory tone, neuroplasticity, and stress related brain function. Across clinical and experimental studies, alterations in T cell subsets, activation states, and trafficking into the CNS are consistently associated with depressive symptoms and treatment response.

A central insight from current research is the context dependent role of distinct T cell populations. Pro inflammatory subsets, particularly Th1 and Th17 cells, amplify neuroinflammation by promoting cytokine release, activating microglia and astrocytes, and suppressing neurogenesis. These processes converge on brain regions critical for mood regulation. In contrast, Tregs and Th2 associated responses limit excessive inflammation and support neural homeostasis. However, excessive or maladaptive Tregs expansion under chronic stress may impair stress adaptability, emphasizing that immune balance rather than uniform suppression is essential.

In addition, sex represents an important biological variable that has not been systematically addressed in most T cell-related depression studies. Given the well-established sex differences in immune activation, hormone regulation, and depression prevalence, future research should incorporate sex-stratified analyses to improve mechanistic clarity and translational relevance.

However, while the evidence supporting T cell involvement in depression is growing, there are notable methodological limitations and reproducibility issues that must be addressed. Much of the work on T cell migration to brain tissue is derived from animal studies in autoimmune conditions such as multiple sclerosis (MS) and EAE, which may not fully translate to primary MDD in humans, particularly in cohorts without immune-mediated comorbidities. The migration patterns and immune activation seen in these autoimmune disorders may differ from those observed in depression, where immune-neural interactions are often more subtle and chronic. Moreover, many clinical studies do not consistently report or stratify immune-mediated comorbidities (e.g., MS, psoriasis, inflammatory bowel disease), which can independently shape T cell subset distribution and function and confound interpretation of “depression-associated” immune signatures. Furthermore, the limited sample sizes, variations in animal models, and differences in study protocols across research groups pose challenges to the reproducibility of findings. Therefore, while T cell-mediated mechanisms in depression hold promise, caution should be exercised in extrapolating results from EAE and MS models to depression without further validation in well-phenotyped human cohorts and mood-disorder–relevant models. From a translational perspective, targeting T cell mediated pathways offers promising opportunities for precision treatment. Strategies aimed at restoring T cell subset balance, modulating cytokine signaling, regulating microbiota driven immune responses, or limiting pathogenic T cell entry into the brain may complement existing antidepressant approaches. Future studies integrating longitudinal immune profiling, neuroimaging, and clinical phenotyping will be essential to define immune based subtypes of depression and guide individualized interventions.

## Glossary

AD: Alzheimer’s disease
ACKR1: Atypical chemokine receptor 1
AJs: Adherens junctions
BBB: Blood–brain barrier
BDNF: Brain-derived neurotrophic factor
BSCB: Blood–spinal cord barrier
CCL20: C–C motif chemokine ligand 20
CCR6: C–C chemokine receptor 6
CIRS: Compensatory immune response system
CNS: Central nervous system
CUMS: Chronic unpredictable mild stress
DsEVs: Dendritic cell–derived small extracellular vesicles
EAE: Experimental autoimmune encephalomyelitis
EVs: Extracellular vesicles
FOXP3: Forkhead box protein P3
FMD: Fasting-mimicking diet
GPCR: G-protein–coupled receptor
HGF: Hepatocyte growth factor
hAMC: Human amnion mesenchymal cells
HPA axis: Hypothalamic–pituitary–adrenal axis
ICAM-1: Intercellular adhesion molecule 1
IDO: Indoleamine 2,3-dioxygenase
IFN-γ: Interferon gamma
IL: Interleukin
IL-17RA: Interleukin-17 receptor A
IRS: Inflammatory response system
JAM: Junctional adhesion molecule
LFA-1: Lymphocyte function–associated antigen 1
MAPK: Mitogen-activated protein kinase
MDD: Major depressive disorder
MMP: Matrix metalloproteinase
MSC-EVs: Mesenchymal stem cell–derived extracellular vesicles
NEEVs: Neuronal-enriched extracellular vesicles
NF-κB: Nuclear factor kappa B
NLRP3: NOD-like receptor family pyrin domain-containing 3
NVU: Neurovascular unit
PBMCs: Peripheral blood mononuclear cells
PD-L1: Programmed death-ligand 1
PSGL-1: P-selectin glycoprotein ligand-1
RORγT: Retinoic acid receptor–related orphan receptor gamma T
SFB: Segmented filamentous bacteria
SSRIs: Selective serotonin reuptake inhibitors
ST2: Suppression of tumorigenicity 2
T-EVs: T cell–derived extracellular vesicles
TGF-β: Transforming growth factor beta
Th cells: T helper cells
TJs: Tight junctions
TNF-α: Tumor necrosis factor alpha
TrkB: Tropomyosin receptor kinase B
Tregs: Regulatory T cells
VCAM-1: Vascular cell adhesion molecule 1
VE-cadherin: Vascular endothelial cadherin
VLA-4: Very late antigen-4
5-HT: 5-hydroxytryptamine (serotonin).
